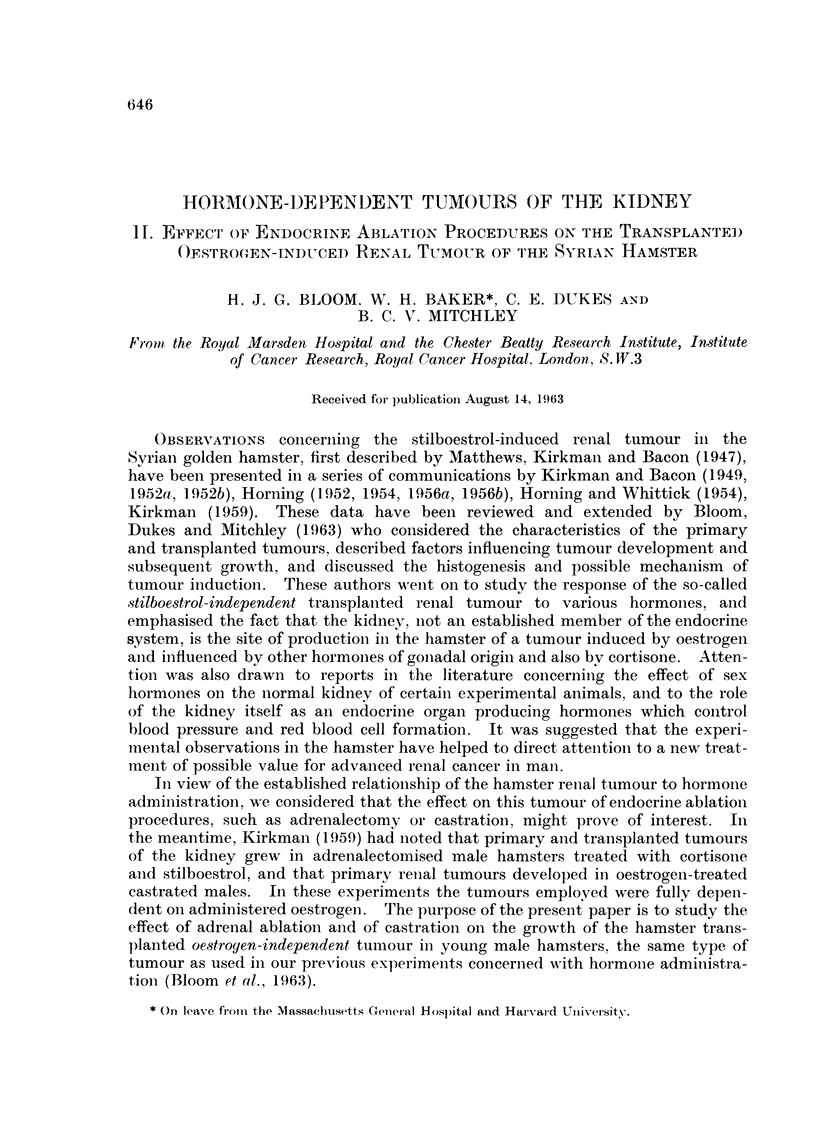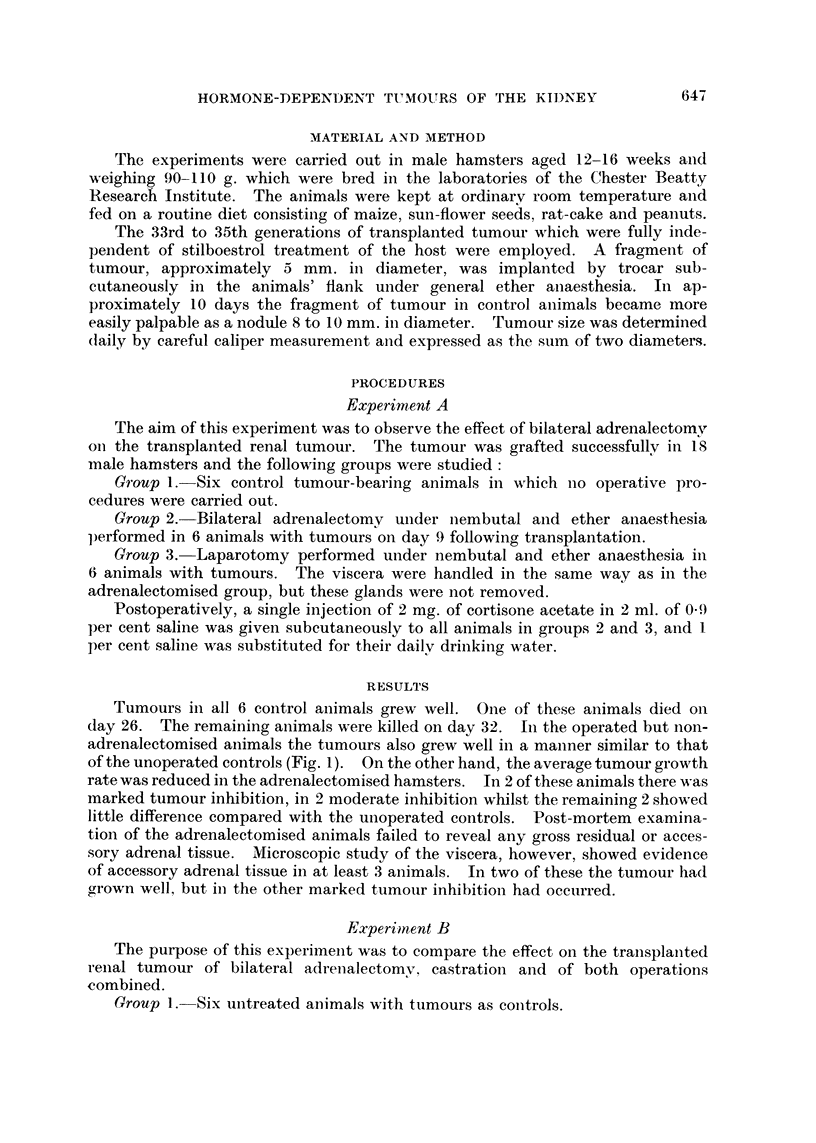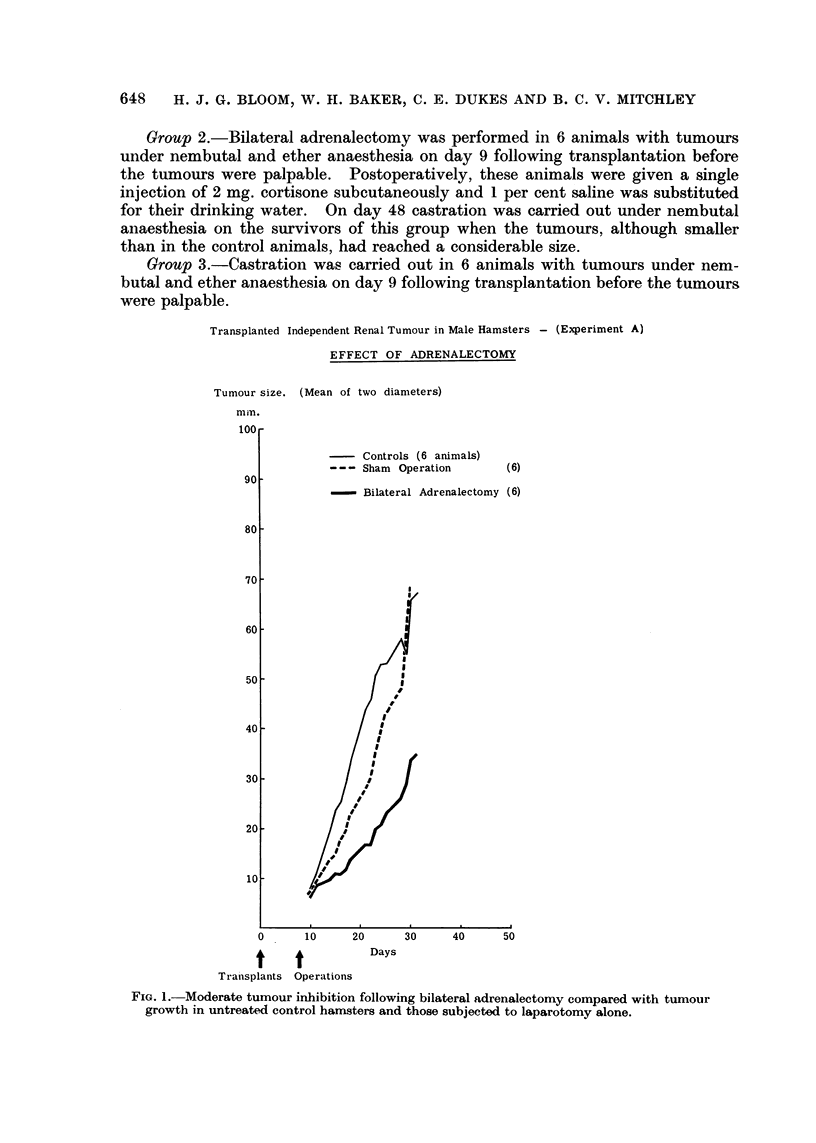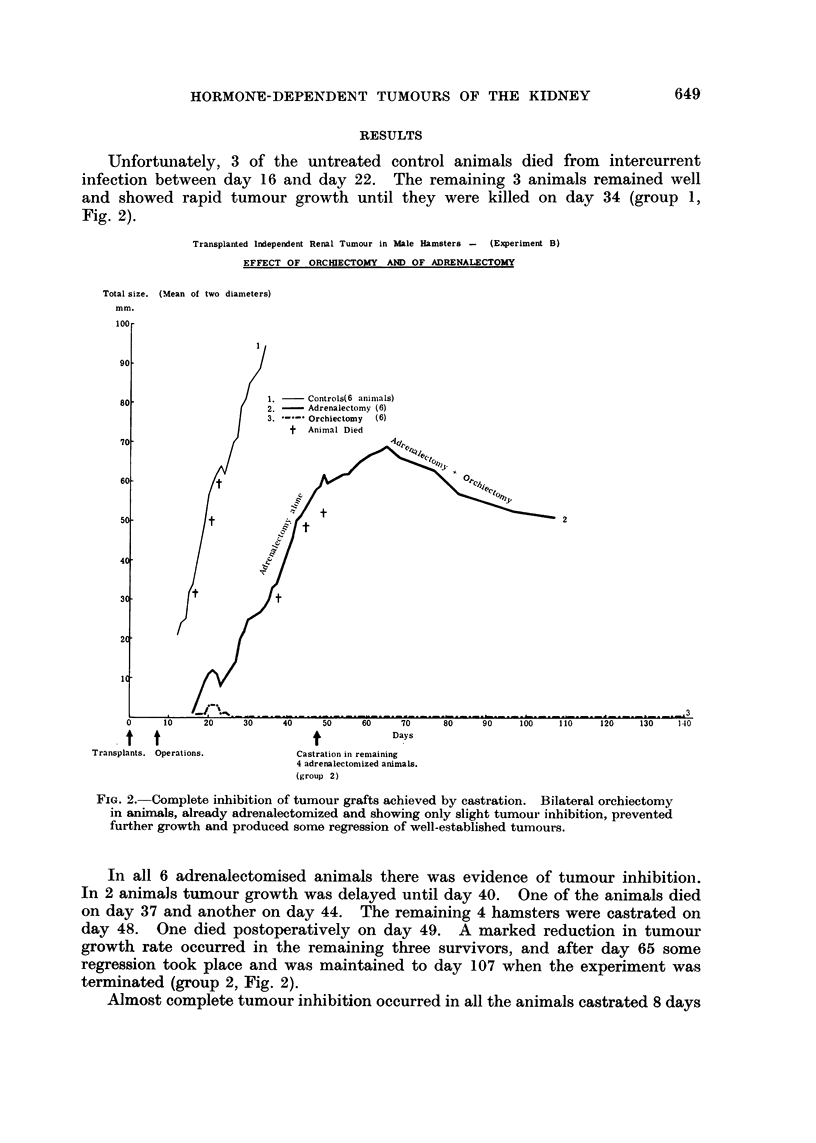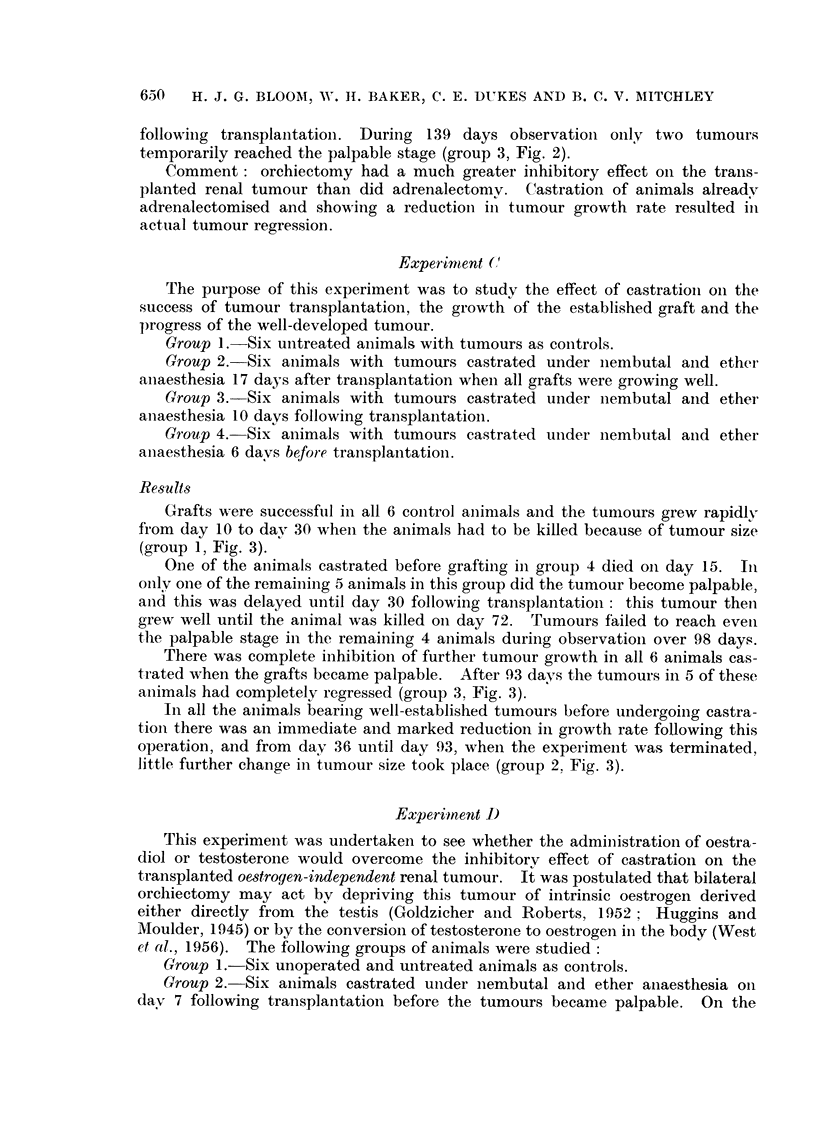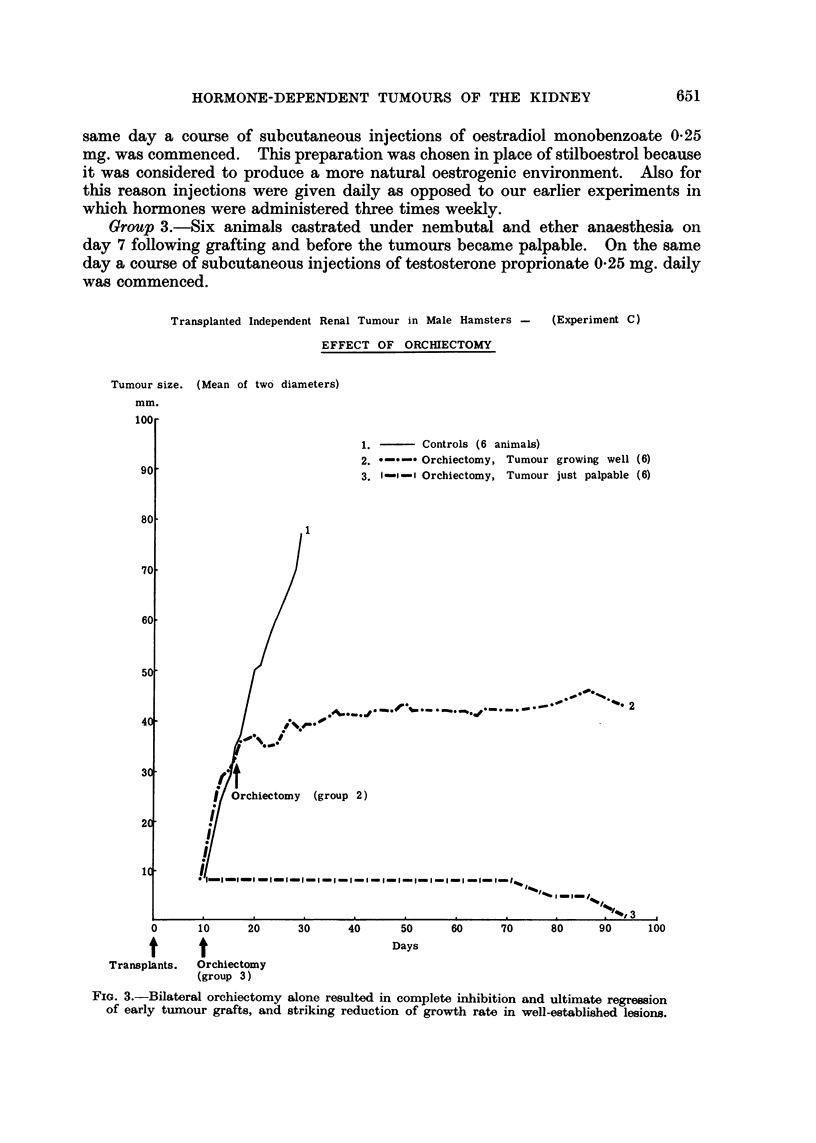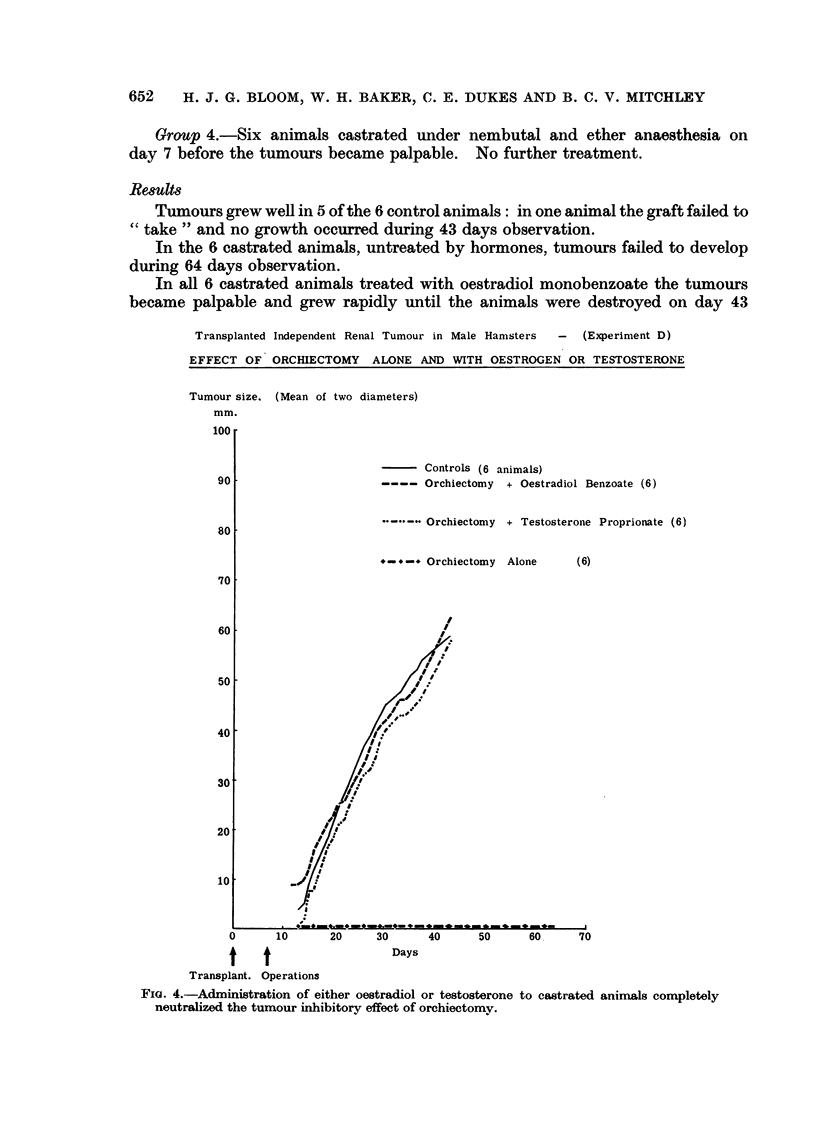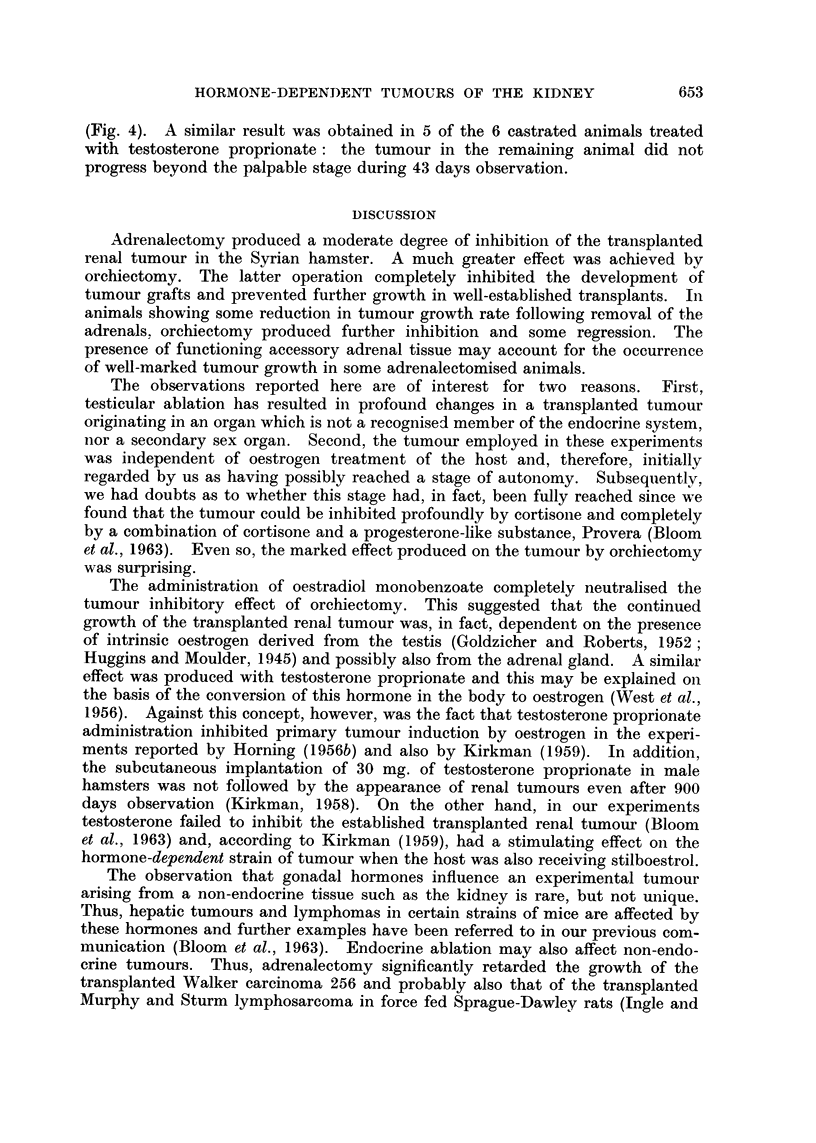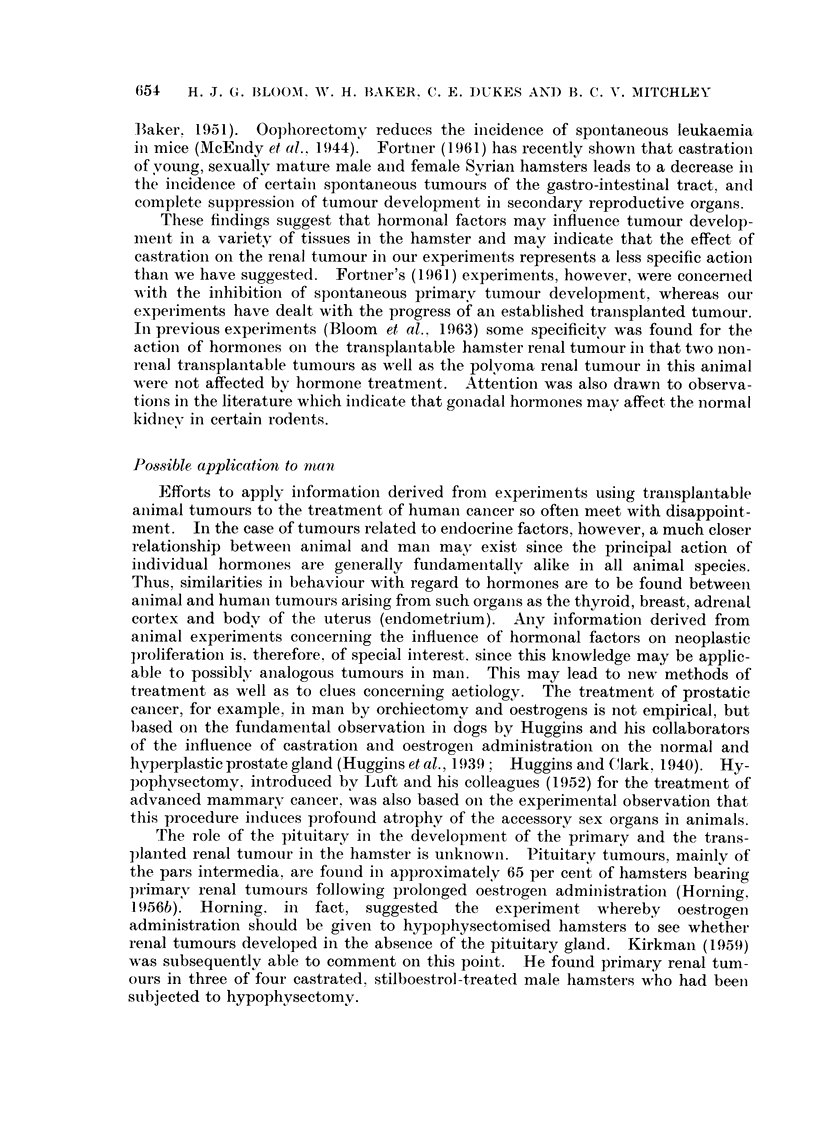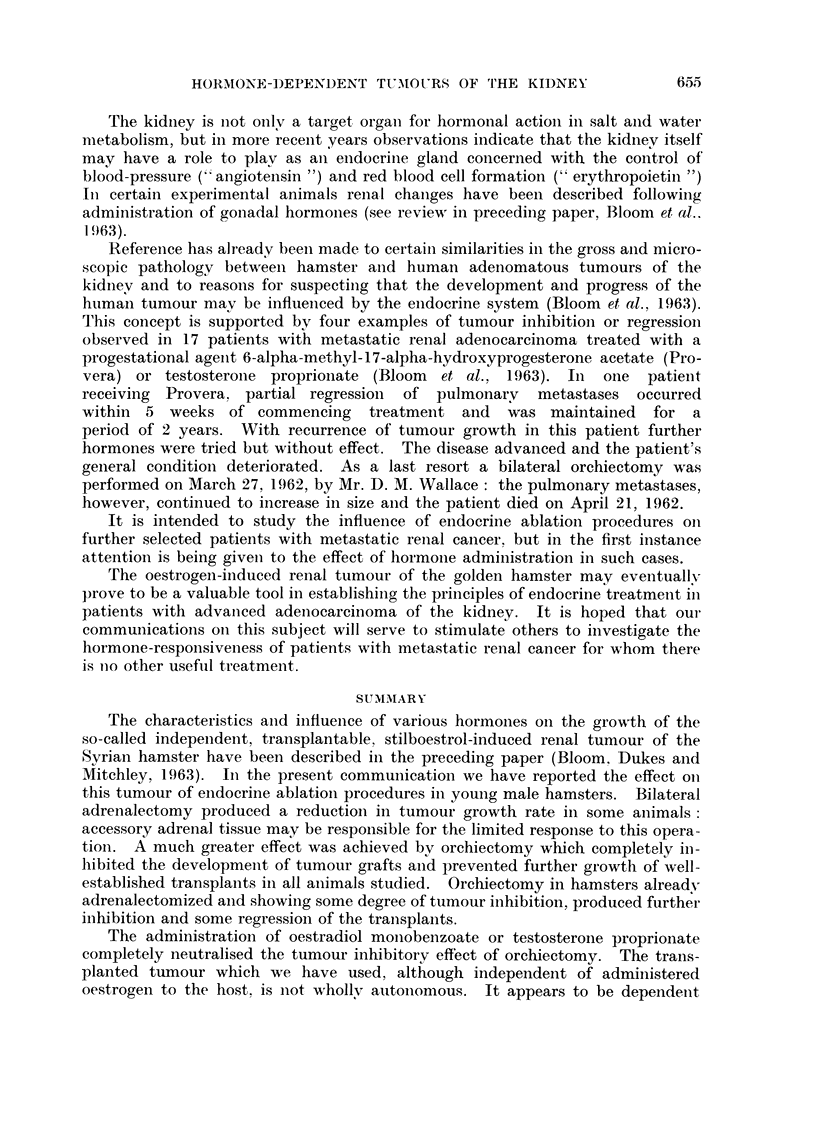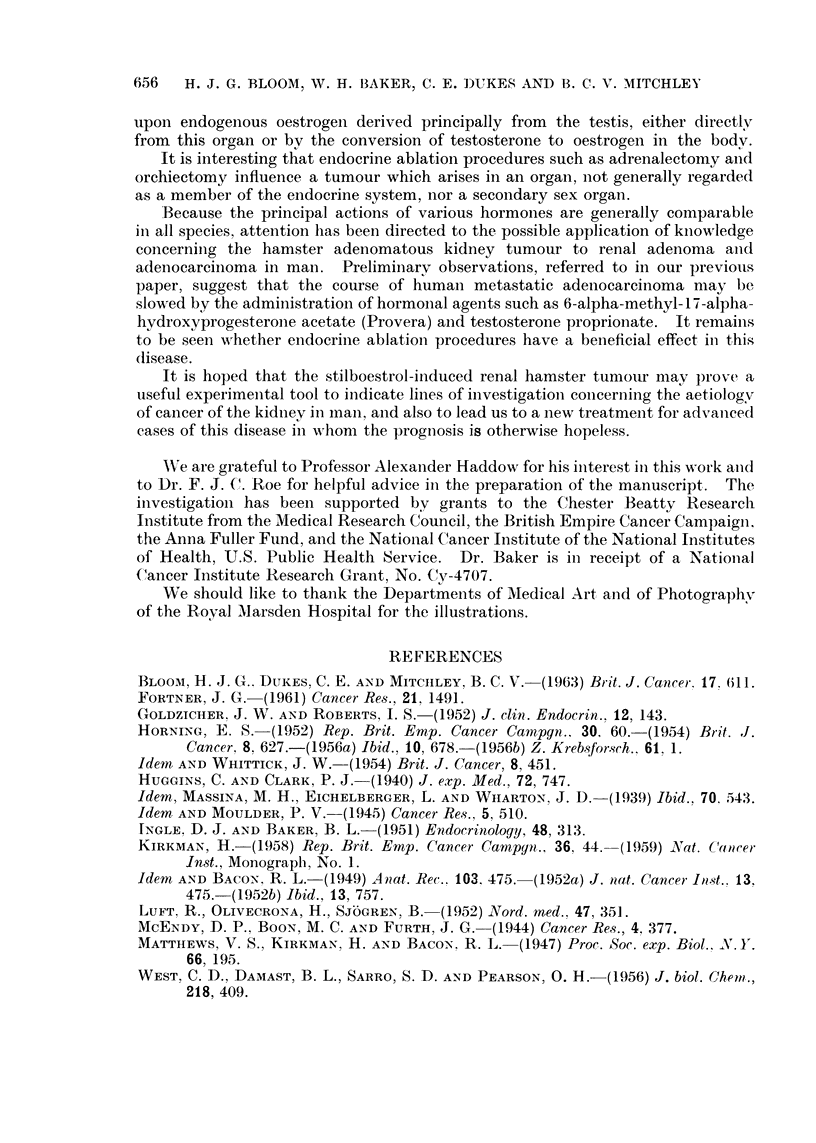# Hormone-Dependent Tumours of the Kidney

**DOI:** 10.1038/bjc.1963.82

**Published:** 1963-12

**Authors:** H. J. G. Bloom, W. H. Baker, C. E. Dukes, B. C. V. Mitchley


### HORMONE-DEPENDENT TUMOURS OF THE KIDNEY

### II. Effect of Endocrine Ablation Procedures on the Transplanted Oestrogen-induced Renal Tumour of the Syrian Hamster

H. J. G. BLOOM, W. H. BAKER*, C. E. DUKES and
B. C. V. MITCHLEY

*From the Royal Marsden Hospital and the Chester Beatty Research Institute, Institute of Cancer Research, Royal Cancer Hospital, London, S.W.3*

Received for publication August 14, 1963

Observations concerning the stilboestrol-induced renal tumour in the Syrian golden hamster, first described by Matthews, Kirkman and Bacon (1947), have been presented in a series of communications by Kirkman and Bacon (1949, 1952*a*, 1952*b*), Horning (1952, 1954, 1956*a*, 1956*b*), Horning and Whittick (1954), Kirkman (1959). These data have been reviewed and extended by Bloom, Dukes and Mitchley (1963) who considered the characteristics of the primary and transplanted tumours, described factors influencing tumour development and subsequent growth, and discussed the histogenesis and possible mechanism of tumour induction. These authors went on to study the response of the so-called *stilboestrol-independent* transplanted renal tumour to various hormones, and emphasised the fact that the kidney, not an established member of the endocrine system, is the site of production in the hamster of a tumour induced by oestrogen and influenced by other hormones of gonadal origin and also by cortisone. Attention was also drawn to reports in the literature concerning the effect of sex hormones on the normal kidney of certain experimental animals, and to the role of the kidney itself as an endocrine organ producing hormones which control blood pressure and red blood cell formation. It was suggested that the experimental observations in the hamster have helped to direct attention to a new treatment of possible value for advanced renal cancer in man.

In view of the established relationship of the hamster renal tumour to hormone administration, we considered that the effect on this tumour of endocrine ablation procedures, such as adrenalectomy or castration, might prove of interest. In the meantime, Kirkman (1959) had noted that primary and transplanted tumours of the kidney grew in adrenalectomised male hamsters treated with cortisone and stilboestrol, and that primary renal tumours developed in oestrogen-treated castrated males. In these experiments the tumours employed were fully dependent on administered oestrogen. The purpose of the present paper is to study the effect of adrenal ablation and of castration on the growth of the hamster transplanted *oestrogen-independent* tumour in young male hamsters, the same type of tumour as used in our previous experiments concerned with hormone administration (Bloom *et al.*, 1963).

* On leave from the Massachusetts General Hospital and Harvard University.

### MATERIAL AND METHOD

The experiments were carried out in male hamsters aged 12–16 weeks and weighing 90–110 g. which were bred in the laboratories of the Chester Beatty Research Institute. The animals were kept at ordinary room temperature and fed on a routine diet consisting of maize, sun-flower seeds, rat-cake and peanuts.

The 33rd to 35th generations of transplanted tumour which were fully independent of stilboestrol treatment of the host were employed. A fragment of tumour, approximately 5 mm. in diameter, was implanted by trocar subcutaneously in the animals' flank under general ether anaesthesia. In approximately 10 days the fragment of tumour in control animals became more easily palpable as a nodule 8 to 10 mm. in diameter. Tumour size was determined daily by careful caliper measurement and expressed as the sum of two diameters.

### PROCEDURES

### *Experiment A*

The aim of this experiment was to observe the effect of bilateral adrenalectomy on the transplanted renal tumour. The tumour was grafted successfully in 18 male hamsters and the following groups were studied :

*Group* 1.—Six control tumour-bearing animals in which no operative procedures were carried out.

*Group* 2.—Bilateral adrenalectomy under nembutal and ether anaesthesia performed in 6 animals with tumours on day 9 following transplantation.

*Group* 3.—Laparotomy performed under nembutal and ether anaesthesia in 6 animals with tumours. The viscera were handled in the same way as in the adrenalectomised group, but these glands were not removed.

Postoperatively, a single injection of 2 mg. of cortisone acetate in 2 ml. of 0·9 per cent saline was given subcutaneously to all animals in groups 2 and 3, and 1 per cent saline was substituted for their daily drinking water.

### RESULTS

Tumours in all 6 control animals grew well. One of these animals died on day 26. The remaining animals were killed on day 32. In the operated but non-adrenalectomised animals the tumours also grew well in a manner similar to that of the unoperated controls (Fig. 1). On the other hand, the average tumour growth rate was reduced in the adrenalectomised hamsters. In 2 of these animals there was marked tumour inhibition, in 2 moderate inhibition whilst the remaining 2 showed little difference compared with the unoperated controls. Post-mortem examination of the adrenalectomised animals failed to reveal any gross residual or accessory adrenal tissue. Microscopic study of the viscera, however, showed evidence of accessory adrenal tissue in at least 3 animals. In two of these the tumour had grown well, but in the other marked tumour inhibition had occurred.

### *Experiment B*

The purpose of this experiment was to compare the effect on the transplanted renal tumour of bilateral adrenalectomy, castration and of both operations combined.

*Group* 1.—Six untreated animals with tumours as controls.

*Group* 2.—Bilateral adrenalectomy was performed in 6 animals with tumours under nembutal and ether anaesthesia on day 9 following transplantation before the tumours were palpable. Postoperatively, these animals were given a single injection of 2 mg. cortisone subcutaneously and 1 per cent saline was substituted for their drinking water. On day 48 castration was carried out under nembutal anaesthesia on the survivors of this group when the tumours, although smaller than in the control animals, had reached a considerable size.

*Group* 3.—Castration was carried out in 6 animals with tumours under nembutal and ether anaesthesia on day 9 following transplantation before the tumours were palpable.

FIG. 1.—Moderate tumour inhibition following bilateral adrenalectomy compared with tumour growth in untreated control hamsters and those subjected to laparotomy alone.

### RESULTS

Unfortunately, 3 of the untreated control animals died from intercurrent infection between day 16 and day 22. The remaining 3 animals remained well and showed rapid tumour growth until they were killed on day 34 (group 1, Fig. 2).

FIG. 2.—Complete inhibition of tumour grafts achieved by castration. Bilateral orchiectomy in animals, already adrenalectomized and showing only slight tumour inhibition, prevented further growth and produced some regression of well-established tumours.

In all 6 adrenalectomised animals there was evidence of tumour inhibition. In 2 animals tumour growth was delayed until day 40. One of the animals died on day 37 and another on day 44. The remaining 4 hamsters were castrated on day 48. One died postoperatively on day 49. A marked reduction in tumour growth rate occurred in the remaining three survivors, and after day 65 some regression took place and was maintained to day 107 when the experiment was terminated (group 2, Fig. 2).

Almost complete tumour inhibition occurred in all the animals castrated 8 days following transplantation. During 139 days observation only two tumours temporarily reached the palpable stage (group 3, Fig. 2).

Comment : orchiectomy had a much greater inhibitory effect on the transplanted renal tumour than did adrenalectomy. Castration of animals already adrenalectomised and showing a reduction in tumour growth rate resulted in actual tumour regression.

*Experiment C*

The purpose of this experiment was to study the effect of castration on the success of tumour transplantation, the growth of the established graft and the progress of the well-developed tumour.

*Group* 1.—Six untreated animals with tumours as controls.

*Group* 2.—Six animals with tumours castrated under nembutal and ether anaesthesia 17 days after transplantation when all grafts were growing well.

*Group* 3.—Six animals with tumours castrated under nembutal and ether anaesthesia 10 days following transplantation.

*Group* 4.—Six animals with tumours castrated under nembutal and ether anaesthesia 6 days *before* transplantation.

*Results*

Grafts were successful in all 6 control animals and the tumours grew rapidly from day 10 to day 30 when the animals had to be killed because of tumour size (group 1, Fig. 3).

One of the animals castrated before grafting in group 4 died on day 15. In only one of the remaining 5 animals in this group did the tumour become palpable, and this was delayed until day 30 following transplantation : this tumour then grew well until the animal was killed on day 72. Tumours failed to reach even the palpable stage in the remaining 4 animals during observation over 98 days.

There was complete inhibition of further tumour growth in all 6 animals castrated when the grafts became palpable. After 93 days the tumours in 5 of these animals had completely regressed (group 3, Fig. 3).

In all the animals bearing well-established tumours before undergoing castration there was an immediate and marked reduction in growth rate following this operation, and from day 36 until day 93, when the experiment was terminated, little further change in tumour size took place (group 2, Fig. 3).

*Experiment D*

This experiment was undertaken to see whether the administration of oestradiol or testosterone would overcome the inhibitory effect of castration on the transplanted *oestrogen-independent* renal tumour. It was postulated that bilateral orchiectomy may act by depriving this tumour of intrinsic oestrogen derived either directly from the testis (Goldzicher and Roberts, 1952 ; Huggins and Moulder, 1945) or by the conversion of testosterone to oestrogen in the body (West *et al.*, 1956). The following groups of animals were studied :

*Group* 1.—Six unoperated and untreated animals as controls.

*Group* 2.—Six animals castrated under nembutal and ether anaesthesia on day 7 following transplantation before the tumours became palpable. On the same day a course of subcutaneous injections of oestradiol monobenzoate 0·25 mg. was commenced. This preparation was chosen in place of stilboestrol because it was considered to produce a more natural oestrogenic environment. Also for this reason injections were given daily as opposed to our earlier experiments in which hormones were administered three times weekly.

*Group* 3.—Six animals castrated under nembutal and ether anaesthesia on day 7 following grafting and before the tumours became palpable. On the same day a course of subcutaneous injections of testosterone proprionate 0·25 mg. daily was commenced.

Transplanted Independent Renal Tumour in Male Hamsters — (Experiment C)

EFFECT OF ORCHIECTOMY

FIG. 3.—Bilateral orchiectomy alone resulted in complete inhibition and ultimate regression of early tumour grafts, and striking reduction of growth rate in well-established lesions.

*Group* 4.—Six animals castrated under nembutal and ether anaesthesia on day 7 before the tumours became palpable. No further treatment.

*Results*

Tumours grew well in 5 of the 6 control animals : in one animal the graft failed to " take " and no growth occurred during 43 days observation.

In the 6 castrated animals, untreated by hormones, tumours failed to develop during 64 days observation.

In all 6 castrated animals treated with oestradiol monobenzoate the tumours became palpable and grew rapidly until the animals were destroyed on day 43

FIG. 4.—Administration of either oestradiol or testosterone to castrated animals completely neutralized the tumour inhibitory effect of orchiectomy.

(Fig. 4). A similar result was obtained in 5 of the 6 castrated animals treated with testosterone proprionate : the tumour in the remaining animal did not progress beyond the palpable stage during 43 days observation.

### DISCUSSION

Adrenalectomy produced a moderate degree of inhibition of the transplanted renal tumour in the Syrian hamster. A much greater effect was achieved by orchiectomy. The latter operation completely inhibited the development of tumour grafts and prevented further growth in well-established transplants. In animals showing some reduction in tumour growth rate following removal of the adrenals, orchiectomy produced further inhibition and some regression. The presence of functioning accessory adrenal tissue may account for the occurrence of well-marked tumour growth in some adrenalectomised animals.

The observations reported here are of interest for two reasons. First, testicular ablation has resulted in profound changes in a transplanted tumour originating in an organ which is not a recognised member of the endocrine system, nor a secondary sex organ. Second, the tumour employed in these experiments was independent of oestrogen treatment of the host and, therefore, initially regarded by us as having possibly reached a stage of autonomy. Subsequently, we had doubts as to whether this stage had, in fact, been fully reached since we found that the tumour could be inhibited profoundly by cortisone and completely by a combination of cortisone and a progesterone-like substance, Provera (Bloom *et al.*, 1963). Even so, the marked effect produced on the tumour by orchiectomy was surprising.

The administration of oestradiol monobenzoate completely neutralised the tumour inhibitory effect of orchiectomy. This suggested that the continued growth of the transplanted renal tumour was, in fact, dependent on the presence of intrinsic oestrogen derived from the testis (Goldzicher and Roberts, 1952 ; Huggins and Moulder, 1945) and possibly also from the adrenal gland. A similar effect was produced with testosterone proprionate and this may be explained on the basis of the conversion of this hormone in the body to oestrogen (West *et al.*, 1956). Against this concept, however, was the fact that testosterone proprionate administration inhibited primary tumour induction by oestrogen in the experiments reported by Horning (1956*b*) and also by Kirkman (1959). In addition, the subcutaneous implantation of 30 mg. of testosterone proprionate in male hamsters was not followed by the appearance of renal tumours even after 900 days observation (Kirkman, 1958). On the other hand, in our experiments testosterone failed to inhibit the established transplanted renal tumour (Bloom *et al.*, 1963) and, according to Kirkman (1959), had a stimulating effect on the hormone-*dependent* strain of tumour when the host was also receiving stilboestrol.

The observation that gonadal hormones influence an experimental tumour arising from a non-endocrine tissue such as the kidney is rare, but not unique. Thus, hepatic tumours and lymphomas in certain strains of mice are affected by these hormones and further examples have been referred to in our previous communication (Bloom *et al.*, 1963). Endocrine ablation may also affect non-endocrine tumours. Thus, adrenalectomy significantly retarded the growth of the transplanted Walker carcinoma 256 and probably also that of the transplanted Murphy and Sturm lymphosarcoma in force fed Sprague-Dawley rats (Ingle and

Baker, 1951). Oophorectomy reduces the incidence of spontaneous leukaemia in mice (McEndy *et al.*, 1944). Fortner (1961) has recently shown that castration of young, sexually mature male and female Syrian hamsters leads to a decrease in the incidence of certain spontaneous tumours of the gastro-intestinal tract, and complete suppression of tumour development in secondary reproductive organs.

These findings suggest that hormonal factors may influence tumour development in a variety of tissues in the hamster and may indicate that the effect of castration on the renal tumour in our experiments represents a less specific action than we have suggested. Fortner's (1961) experiments, however, were concerned with the inhibition of spontaneous primary tumour development, whereas our experiments have dealt with the progress of an established transplanted tumour. In previous experiments (Bloom *et al.*, 1963) some specificity was found for the action of hormones on the transplantable hamster renal tumour in that two non-renal transplantable tumours as well as the polyoma renal tumour in this animal were not affected by hormone treatment. Attention was also drawn to observations in the literature which indicate that gonadal hormones may affect the normal kidney in certain rodents.

*Possible application to man*

Efforts to apply information derived from experiments using transplantable animal tumours to the treatment of human cancer so often meet with disappointment. In the case of tumours related to endocrine factors, however, a much closer relationship between animal and man may exist since the principal action of individual hormones are generally fundamentally alike in all animal species. Thus, similarities in behaviour with regard to hormones are to be found between animal and human tumours arising from such organs as the thyroid, breast, adrenal cortex and body of the uterus (endometrium). Any information derived from animal experiments concerning the influence of hormonal factors on neoplastic proliferation is, therefore, of special interest, since this knowledge may be applicable to possibly analogous tumours in man. This may lead to new methods of treatment as well as to clues concerning aetiology. The treatment of prostatic cancer, for example, in man by orchiectomy and oestrogens is not empirical, but based on the fundamental observation in dogs by Huggins and his collaborators of the influence of castration and oestrogen administration on the normal and hyperplastic prostate gland (Huggins *et al.*, 1939; Huggins and Clark, 1940). Hypophysectomy, introduced by Luft and his colleagues (1952) for the treatment of advanced mammary cancer, was also based on the experimental observation that this procedure induces profound atrophy of the accessory sex organs in animals.

The role of the pituitary in the development of the primary and the transplanted renal tumour in the hamster is unknown. Pituitary tumours, mainly of the pars intermedia, are found in approximately 65 per cent of hamsters bearing primary renal tumours following prolonged oestrogen administration (Horning, 1956*b*). Horning, in fact, suggested the experiment whereby oestrogen administration should be given to hypophysectomised hamsters to see whether renal tumours developed in the absence of the pituitary gland. Kirkman (1959) was subsequently able to comment on this point. He found primary renal tumours in three of four castrated, stilboestrol-treated male hamsters who had been subjected to hypophysectomy.

The kidney is not only a target organ for hormonal action in salt and water metabolism, but in more recent years observations indicate that the kidney itself may have a role to play as an endocrine gland concerned with the control of blood-pressure ("angiotensin") and red blood cell formation ("erythropoietin") In certain experimental animals renal changes have been described following administration of gonadal hormones (see review in preceding paper, Bloom *et al.*, 1963).

Reference has already been made to certain similarities in the gross and microscopic pathology between hamster and human adenomatous tumours of the kidney and to reasons for suspecting that the development and progress of the human tumour may be influenced by the endocrine system (Bloom *et al.*, 1963). This concept is supported by four examples of tumour inhibition or regression observed in 17 patients with metastatic renal adenocarcinoma treated with a progestational agent 6-alpha-methyl-17-alpha-hydroxyprogesterone acetate (Provera) or testosterone proprionate (Bloom *et al.*, 1963). In one patient receiving Provera, partial regression of pulmonary metastases occurred within 5 weeks of commencing treatment and was maintained for a period of 2 years. With recurrence of tumour growth in this patient further hormones were tried but without effect. The disease advanced and the patient's general condition deteriorated. As a last resort a bilateral orchiectomy was performed on March 27, 1962, by Mr. D. M. Wallace : the pulmonary metastases, however, continued to increase in size and the patient died on April 21, 1962.

It is intended to study the influence of endocrine ablation procedures on further selected patients with metastatic renal cancer, but in the first instance attention is being given to the effect of hormone administration in such cases.

The oestrogen-induced renal tumour of the golden hamster may eventually prove to be a valuable tool in establishing the principles of endocrine treatment in patients with advanced adenocarcinoma of the kidney. It is hoped that our communications on this subject will serve to stimulate others to investigate the hormone-responsiveness of patients with metastatic renal cancer for whom there is no other useful treatment.

### SUMMARY

The characteristics and influence of various hormones on the growth of the so-called independent, transplantable, stilboestrol-induced renal tumour of the Syrian hamster have been described in the preceding paper (Bloom, Dukes and Mitchley, 1963). In the present communication we have reported the effect on this tumour of endocrine ablation procedures in young male hamsters. Bilateral adrenalectomy produced a reduction in tumour growth rate in some animals : accessory adrenal tissue may be responsible for the limited response to this operation. A much greater effect was achieved by orchiectomy which completely inhibited the development of tumour grafts and prevented further growth of well-established transplants in all animals studied. Orchiectomy in hamsters already adrenalectomized and showing some degree of tumour inhibition, produced further inhibition and some regression of the transplants.

The administration of oestradiol monobenzoate or testosterone proprionate completely neutralised the tumour inhibitory effect of orchiectomy. The transplanted tumour which we have used, although independent of administered oestrogen to the host, is not wholly autonomous. It appears to be dependent upon endogenous oestrogen derived principally from the testis, either directly from this organ or by the conversion of testosterone to oestrogen in the body.

It is interesting that endocrine ablation procedures such as adrenalectomy and orchiectomy influence a tumour which arises in an organ, not generally regarded as a member of the endocrine system, nor a secondary sex organ.

Because the principal actions of various hormones are generally comparable in all species, attention has been directed to the possible application of knowledge concerning the hamster adenomatous kidney tumour to renal adenoma and adenocarcinoma in man. Preliminary observations, referred to in our previous paper, suggest that the course of human metastatic adenocarcinoma may be slowed by the administration of hormonal agents such as 6-alpha-methyl-17-alpha-hydroxyprogesterone acetate (Provera) and testosterone proprionate. It remains to be seen whether endocrine ablation procedures have a beneficial effect in this disease.

It is hoped that the stilboestrol-induced renal hamster tumour may prove a useful experimental tool to indicate lines of investigation concerning the aetiology of cancer of the kidney in man, and also to lead us to a new treatment for advanced cases of this disease in whom the prognosis is otherwise hopeless.

We are grateful to Professor Alexander Haddow for his interest in this work and to Dr. F. J. C. Roe for helpful advice in the preparation of the manuscript. The investigation has been supported by grants to the Chester Beatty Research Institute from the Medical Research Council, the British Empire Cancer Campaign, the Anna Fuller Fund, and the National Cancer Institute of the National Institutes of Health, U.S. Public Health Service. Dr. Baker is in receipt of a National Cancer Institute Research Grant, No. Cy-4707.

We should like to thank the Departments of Medical Art and of Photography of the Royal Marsden Hospital for the illustrations.

### REFERENCES

BLOOM, H. J. G., DUKES, C. E. AND MITCHLEY, B. C. V.—(1963) *Brit. J. Cancer*, **17**, 611.

FORTNER, J. G.—(1961) *Cancer Res.*, **21**, 1491.

GOLDZICHER, J. W. AND ROBERTS, I. S.—(1952) *J. clin. Endocrin.*, **12**, 143.

HORNING, E. S.—(1952) *Rep. Brit. Emp. Cancer Campgn.*, **30**, 60.—(1954) *Brit. J. Cancer*, **8**, 627.—(1956*a*) *Ibid.*, **10**, 678.—(1956*b*) *Z. Krebsforsch.*, **61**, 1.

*Idem* AND WHITTICK, J. W.—(1954) *Brit. J. Cancer*, **8**, 451.

HUGGINS, C. AND CLARK, P. J.—(1940) *J. exp. Med.*, **72**, 747.

*Idem*, MASSINA, M. H., EICHELBERGER, L. AND WHARTON, J. D.—(1939) *Ibid.*, **70**, 543.

*Idem* AND MOULDER, P. V.—(1945) *Cancer Res.*, **5**, 510.

INGLE, D. J. AND BAKER, B. L.—(1951) *Endocrinology*, **48**, 313.

KIRKMAN, H.—(1958) *Rep. Brit. Emp. Cancer Campgn.*, **36**, 44.—(1959) *Nat. Cancer Inst.*, Monograph, No. 1.

*Idem* AND BACON, R. L.—(1949) *Anat. Rec.*, **103**, 475.—(1952*a*) *J. nat. Cancer Inst.*, **13**, 475.—(1952*b*) *Ibid.*, **13**, 757.

LUFT, R., OLIVECRONA, H., SJÖGREN, B.—(1952) *Nord. med.*, **47**, 351.

MCENDY, D. P., BOON, M. C. AND FURTH, J. G.—(1944) *Cancer Res.*, **4**, 377.

MATTHEWS, V. S., KIRKMAN, H. AND BACON, R. L.—(1947) *Proc. Soc. exp. Biol.*, *N.Y.* **66**, 195.

WEST, C. D., DAMAST, B. L., SARRO, S. D. AND PEARSON, O. H.—(1956) *J. biol. Chem.*, **218**, 409.